# CD5-positive diffuse large B-cell lymphoma of the uterus: a Case Report with cytologic findings from uterine cavity drainage and literature review

**DOI:** 10.3389/fonc.2026.1744965

**Published:** 2026-04-07

**Authors:** Qinling Yu, Chaoju Fu, Feng Jing, Ying Zeng

**Affiliations:** Department of Pathology, The Thirteenth People’s Hospital of Chongqing, Chongqing, China

**Keywords:** CD5-positive, diffuse large B-cell lymphoma, liquid-based cytology, uterine neoplasms, uterine cavity drainage fluid

## Abstract

**Objective:**

Cases of diffuse large B-cell lymphoma (DLBCL) of the uterus co-expressing CD5 are exceedingly rare and diagnostically challenging due to its nonspecific clinical and radiological features, which often mimic other uterine malignancies. This study retrospectively analyzes the cytopathological and histopathological characteristics of this entity in uterine cavity drainage fluid to facilitate its recognition.

**Methods:**

We analyzed a case of uterine CD5-positive DLBCL by collecting clinical data, imaging findings, cytology from uterine drainage fluid, and histology from endometrial curettage. A review of the pertinent literature on uterine DLBCL was also performed.

**Results:**

A 74-year-old woman presented with a two-week history of abdominal distension and anorexia. Abdominal CT demonstrated uterine enlargement with a large fluid collection and multiple enlarged abdominal lymph nodes. Cytology of the uterine drainage fluid revealed numerous atypical lymphoid cells with large, hyperchromatic nuclei, scant basophilic cytoplasm, and a high nuclear-to-cytoplasmic ratio. Histology of the curettage specimens showed a diffuse infiltrate of large lymphocytes with necrosis. The tumor cells exhibited round, oval, or irregular nuclei, coarse chromatin, and prominent nucleoli. Immunohistochemically, the cells were positive for CD20, CD79α, CD19, PAX5, CD5, Bcl-6, and MUM1, and negative for CD3, CD10, Bcl-2, CD30 and Cyclin D1. *In situ* hybridization for Epstein-Barr virus-encoded small RNA (EBER) was negative. The Ki-67 proliferation index was 90%. These findings supported a diagnosis of CD5-positive DLBCL, non-germinal center B-cell subtype.

**Conclusion:**

Uterus DLBCL expressing CD5 is an exceedingly rare malignancy. Its diagnosis necessitates a comprehensive approach integrating clinical presentation, imaging, cytomorphology, and immunohistochemistry. Increased awareness of the cytological features of lymphoma in uterine drainage fluid is essential to prevent diagnostic oversight.

## 1 Introduction

Diffuse large B-cell lymphoma (DLBCL) is a relatively common and highly heterogeneous type of non-Hodgkin lymphoma (NHL), accounting for approximately 30% to 40% of all NHL cases. However, cases of DLBCL expressing CD5 are relatively rare, constituting only 5% to 10% of all DLBCL cases (1, 2). While extranodal NHL most frequently involves the gastrointestinal tract and central nervous system, primary involvement of the female reproductive system is uncommon, accounting for only 0.2% to 1.1% of extranodal NHL cases. Primary uterine NHL is particularly rare, representing approximately 14% of female reproductive system NHL (3, 4). Uterine DLBCL poses considerable diagnostic difficulties owing to its rarity and nonspecific symptom profile, which overlaps with more common entities like endometrial carcinoma. This is particularly true for diagnoses based on cytological examination of uterine cavity drainage fluid. We herein report a case of uterine CD5-positive DLBCL in an elderly female patient. This study analyzes the cytological findings of uterine cavity drainage fluid, histopathological features of endometrial curettage, ultrasonographic imaging, and laboratory findings in relation to the diagnosis and management of uterine DLBCL. Additionally, it provides a comprehensive review of the literature of uterine DLBCL.

## 2 Materials and methods

### 2.1 Materials

This study is based on the clinical, imaging, cytological, and histopathological data from a case of uterine DLBCL. A comprehensive review of the literature pertaining to uterine DLBCL was also performed. A systematic literature search was performed in the PubMed and CNKI databases for articles published from 2005 to 2025. The search strategy utilized keywords including “Uterus,” “Diffuse Large B-Cell Lymphoma,” and “CD5-positive Diffuse Large B-Cell Lymphoma.” A total of 32 relevant publications were identified, comprising 18 in English and 14 in Chinese.

### 2.2 Methods

The clinically submitted uterine cavity drainage fluid was promptly centrifuged at 2200 rpm for 10 minutes. After centrifugation, the supernatant was discarded, and 5 mL of cervical cell preservation fluid (Hangzhou Healthsky Biotechnology, China.) was added for mixing and preservation. After 30 minutes of preservation, the sample was centrifuged again at 2200 rpm for 10 minutes. The supernatant was discarded, 5 mL of cell diluent was added, and the sample was mixed by pipetting. Subsequently, 1.5 mL of the sample was used to prepare slides via the manual sedimentation-based liquid-based thin-layer cytology technique. The slides were fixed with 95% ethanol, stained using the Papanicolaou method, and examined under a light microscope.

The clinically submitted uterine curettage tissue specimens were processed through fixation, dehydration, clearing, paraffin infiltration, embedding, sectioning, and hematoxylin and eosin (H&E) staining for microscopic examination.

Immunohistochemical (IHC) staining was performed using the fully automated Roche Benchmark Ultra platform. The primary antibodies used included CD5, Ki-67, CD20, CD30, CD79α, CD19, PAX5, Bcl-6, MUM1, CD3, CD10, Bcl-2, Cyclin D1, and C-Myc. All primary antibodies were procured from ZSGB-BIO (Zhongshan Golden Bridge Biotechnology, China). The detection of Epstein-Barr virus-encoded small RNA (EBER) was performed via *in situ* hybridization (ISH), whereas the status of the *C-myc* gene was determined by fluorescence *in situ* hybridization (FISH). The staining procedure was strictly conducted according to the manufacturer’s instructions for each reagent.

## 3 Results

### 3.1 Clinical data

**Figure 1:**
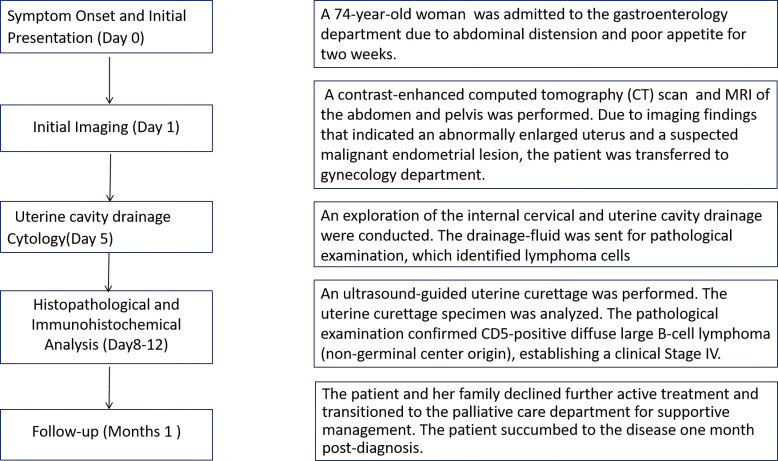
The patient’s diagnostic and therapeutic pathway.

A 74-year-old woman was admitted to the gastroenterology department due to abdominal distension and poor appetite for two weeks(Day0). She also experienced concomitant symptoms of fatigue, a bitter taste in the mouth (referred to as bitter dysgeusia), exertional dyspnea, and weight loss. Notably, she denied any chills, rigors, fever, vaginal bleeding, or other discomforts. Due to imaging findings that indicated an abnormally enlarged uterus and a suspected malignant endometrial lesion, the patient was transferred to gynecology department(Day1). A diagnostic dilation and curettage (D&C) could have provided a definitive diagnosis but was not immediately performed due to the patient’s persistent fever, cervical atrophy, and adhesions. Instead, on Day 5, an exploration of the internal cervical and uterine cavity drainage were conducted. The drainage-fluid was sent for pathological examination, which identified lymphoma cells. To further characterize the lymphoma, an ultrasound-guided uterine curettage was performed on Day 8. The postoperative pathological examination confirmed CD5-positive DLBCL (non-germinal center B-cell, non-GCB), establishing a clinical Stage IV. After multiple discussions, the patient and her family declined further active treatment and transitioned to the palliative care department for supportive management, including antispasmodics, analgesics, meropenem, ornidazole, and fluid replacement with nutritional support. Ultimately, on Day 13, the patient’s family strongly requested discharge for home-based care. The patient’s diagnostic and therapeutic pathway is summarized in **Figure 1**.

Her gynecological history revealed regular menstrual cycles with moderate flow, no dysmenorrhea, and normal leukorrhea before menopause. Her obstetric history was gravida 3, para 3 (G3P3), and she underwent menopause at the age of 45.

### 3.2 Physical examination

Physical examination revealed a fatigued, pale patient without superficial lymphadenopathy. The right lower quadrant was distended, with deep tenderness and a palpable firm mass in the mid- and right lower abdomen. After admission, the patient experienced recurrent spiking fevers, with temperatures peaking from 38 °C to 39 °C.

### 3.3 Laboratory findings

Notable laboratory abnormalities were observed, including notably elevated lactate dehydrogenase (LDH) at 1156.8 U/L and a remarkably high C-reactive protein (CRP) level of 73.57 mg/L. Additionally, carbohydrate antigen 125 (CA-125) was increased at 56.61 U/mL, and α-hydroxybutyrate dehydrogenase (α-HBDH) was elevated at 771.8 U/L.

#### 3.3.1 Hematological parameters

The complete blood count revealed pancytopenia, characterized by a low red blood cell count (3.50 × 10¹²/L), hemoglobin (92 g/L), and platelet count (99 × 10^9^/L). The leukocyte differential showed lymphocytopenia (absolute count 0.85 × 10^9^/L), monocytosis (absolute count 0.64 × 10^9^/L), and an elevated neutrophil percentage of 76.50%. The total leukocyte and absolute neutrophil counts were normal.

#### 3.3.2 Other laboratory findings

Serum levels of troponin, B-type natriuretic peptide (BNP), carcinoembryonic antigen (CEA), Carbohydrate Antigen 19-9 (CA19-9), alpha-fetoprotein (AFP), and procalcitonin were all within normal limits. Human papillomavirus (HPV) testing yielded a negative result.

### 3.4 Imaging findings

#### 3.4.1 Transvaginal and transabdominal ultrasonography

Ultrasound demonstrated marked uterine enlargement with heterogeneous, hypervascular myometrium, massive intrauterine fluid collection with adhesions, and irregular endometrial thickening with suspicious echogenic nodules.

Ultrasound revealed an enlarged postmenopausal uterus (12.5 × 6.6 × 7.9 cm) with a smooth serosal surface, heterogeneous myometrial echotexture, and marked hypervascularity on Color Doppler Flow Imaging (CDFI). The uterine cavity contained a large fluid collection (11.5 × 4.1 cm) with internal adhesions and irregular endometrial thickening (up to 0.92 cm single-layer). Several hyperechoic endometrial nodules were noted, the largest measuring 2.87 × 0.84 cm, with scant punctate blood flow on CDFI. The bilateral ovaries were not visualized. Both adnexa demonstrated cystic, sausage-shaped structures with septations and folds, consistent with hydrosalpinx (left: 1.69 cm; right: 1.56 cm in diameter), showing no internal blood flow but punctate wall signals on CDFI. An irregular anechoic area (1.9 × 2.4 cm) was observed in the pouch of Douglas, indicating pelvic free fluid.

#### 3.4.2 Computed tomography findings

**Figure 2:**
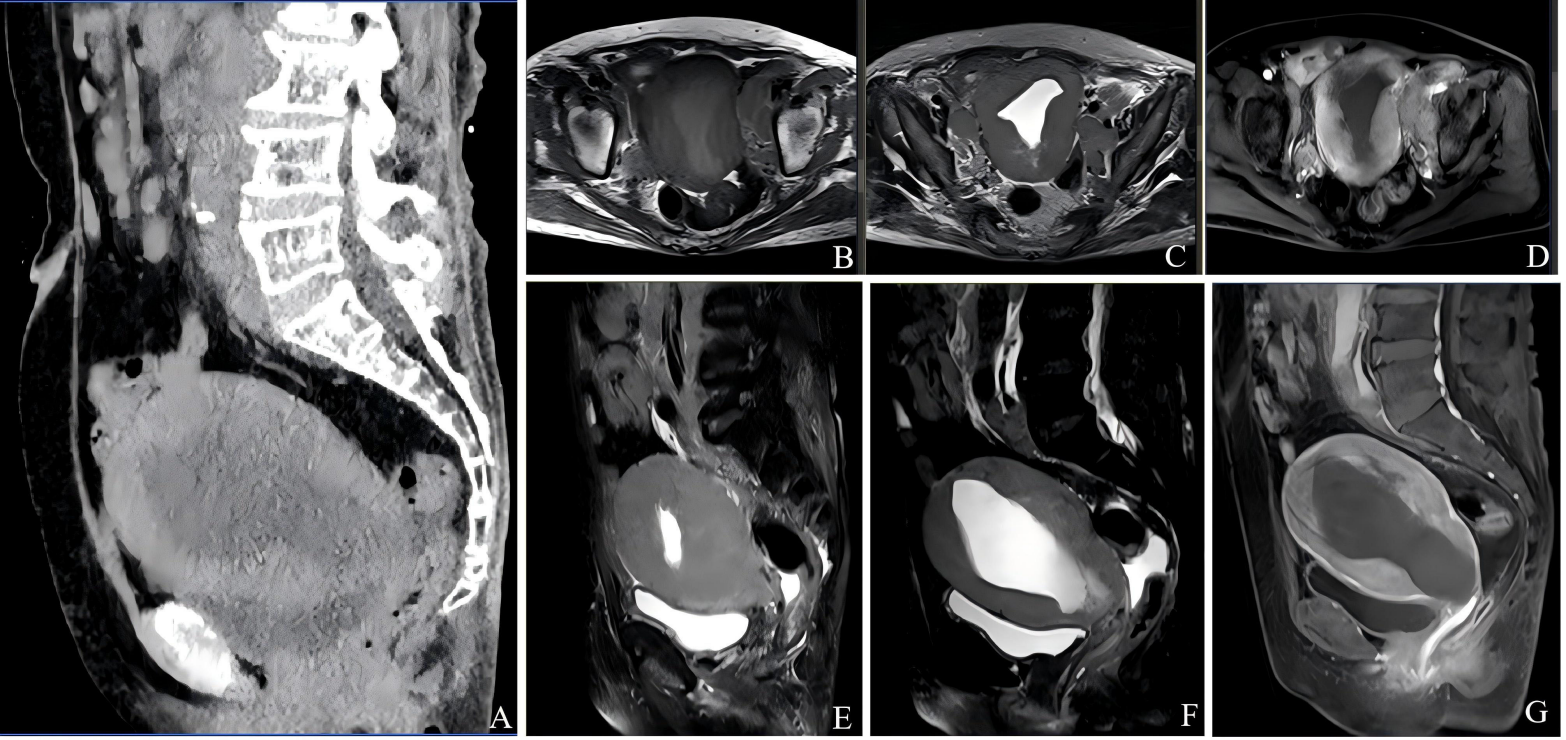
Imaging findings of uterine diffuse large B-cell lymphoma. **(A)** Contrast-enhanced abdominal CT reveals an enlarged uterus containing a large fluid collection (approximately 4.9 cm × 11.2 cm in the sagittal plane; attenuation ~15 HU). Multiple enlarged lymph nodes are present in the mesenteric root, para-aortic region, and bilateral iliac areas. **(B–G)** Pelvic MRI demonstrates uterine enlargement with inhomogeneous myometrial thickening and intrauterine fluid. **(B)** Axial T1-weighted image (T1WI) shows hypointense signal. **(C, E, F)** T2-weighted images (T2WI) **(C)** axial; **(E, F)** sagittal show slightly hypointense signal with patchy areas of T2WI hyperintensity. **(D, G)** Post-contrast images **(D)** axial; **(G)** sagittal demonstrate heterogeneous enhancement of the uterine wall.

Non-contrast CT demonstrated an enlarged uterus containing a sizable fluid-attenuation collection within the endometrial cavity, measuring approximately 11.2 × 4.9 cm in the sagittal plane, with a mean density of 15 Hounsfield Units. Multiple enlarged lymph nodes were noted in the mesenteric root, para-aortic region, and along the bilateral iliac vessels (**Figure 2A**). A small volume of ascites and pelvic free fluid was also present.

#### 3.4.3 Magnetic resonance imaging findings

MRI Findings are highly indicative of Endometrial Carcinoma with suspected cervical stromal involvement and extensive lymph node metastases (mesenteric, retroperitoneal, and bilateral iliac), consistent with FIGO Stage IIIC disease. And large intrauterine fluid collection with associated intracavitary adhesions.

Contrast-enhanced MRI revealed marked uterine enlargement with a large, irregular endometrial mass measuring approximately 13.6 cm × 9.9 cm × 7.9 cm. The lesion was T1 hypointense (**Figure 2B**) and mildly T2 hypointense (**Figures 2C, E, F**), with restricted diffusion manifesting as marked hyperintensity on DWI. Internal patchy T2 hyperintensities were noted. Following contrast administration, the lesion demonstrated progressive, heterogeneous, and marked enhancement (**Figures 2D, G**).The mass demonstrated an ill-defined border with the atrophic cervix, and the junctional zone was obliterated. The myometrium was attenuated and poorly visualized. The uterine cavity contained a large fluid collection with linear, low-signal structures on all sequences, suggestive of adhesions.

Multiple enlarged lymph nodes, notably in the mesenteric root, retroperitoneum, and along the bilateral iliac vessels, showed marked hyperintensity on DWI and formed confluent masses. The largest para-aortic node (approximately 4.9 cm × 3.4 cm) exhibited pronounced enhancement. Inflammatory exudative changes and edema in the lower abdomen and pelvis, with ascites.

### 3.5 Pathological examination

#### 3.5.1 Uterine cavity drainage fluid and cervical liquid-based thin-layer cytology

Cervical Liquid-Based Thin-Layer Cytology revealed atypical squamous cells of undetermined significance (ASC-US).

**Figure 3:**
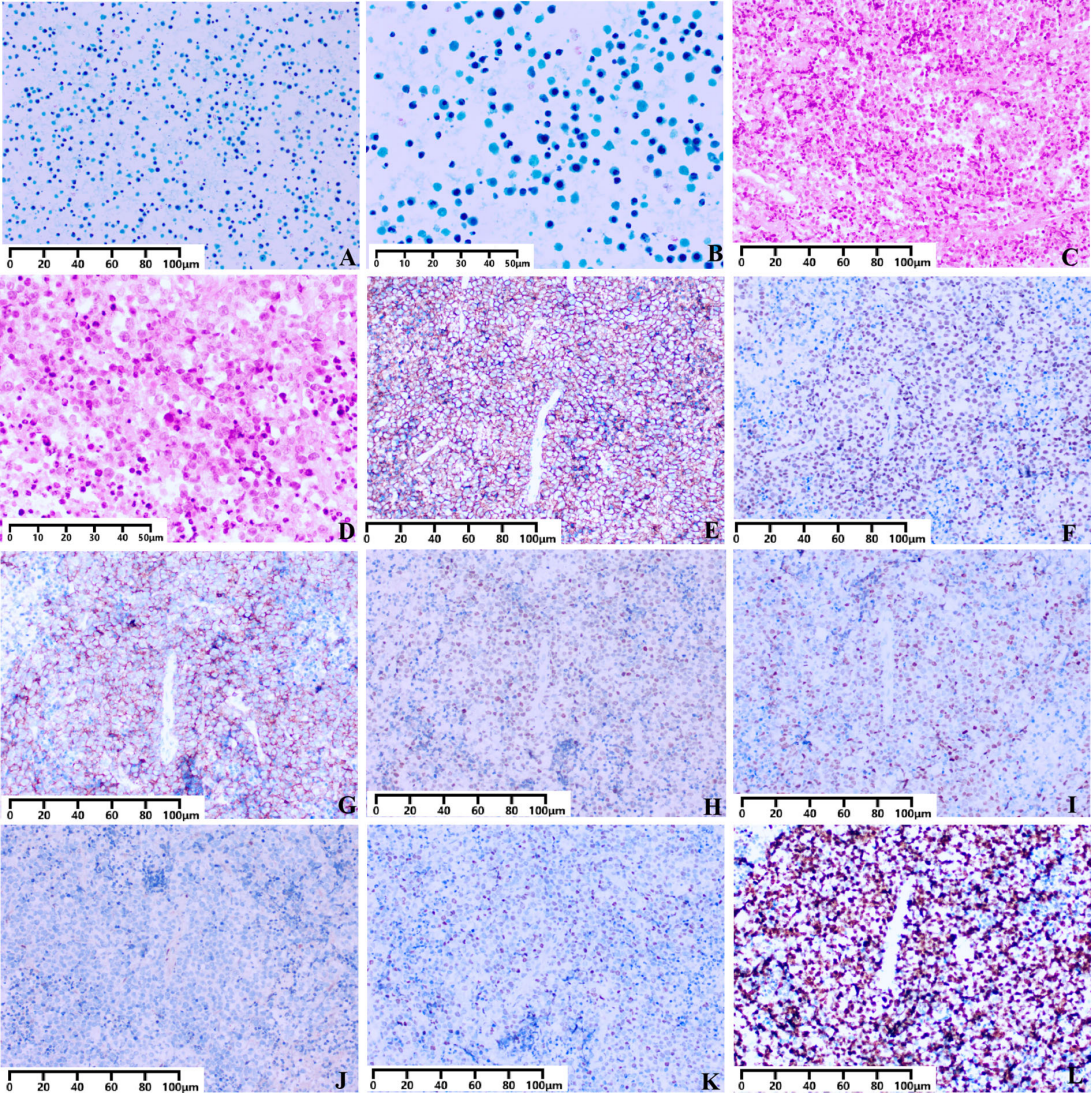
Cytological and histopathological features of uterine diffuse large B-cell lymphoma. **(A, B)** Liquid-based cytology of uterine cavity drainage. **(A)** Medium-power view reveals necrotic cellular debris and scattered enlarged lymphoid cells (Pap stain, ×200; Scale bar: 100 µm). **(B)** High-power view shows large atypical cells with round, oval, or irregular nuclei, a high nuclear-to-cytoplasmic ratio, coarse chromatin, and conspicuous mitotic figures and apoptotic bodies (Pap stain, ×400; Scale bar: 50 µm). **(C, D)** Histological findings of endometrial curettage. **(C)** The tumor cells diffusely infiltrate the endometrial tissue, exhibiting a characteristic “starry-sky” pattern (H&E stain, ×200; Scale bar: 100 µm). **(D)** High-power view demonstrates a monotonous population of large tumor cells with round to irregular nuclei, a high nuclear-to-cytoplasmic ratio, coarse chromatin, 1–3 nucleoli, and frequent mitotic figures and apoptosis (H&E stain, ×400; Scale bar: 50 µm). **(E–L)** Immunohistochemical staining profile. The tumor cells are diffusely positive for B-cell markers CD20 **(E)** and PAX5 **(F)**, and are also positive for CD5 **(G)**, Bcl-6 **(H)**, and MUM1 **(I)**. The neoplastic cells are negative for Cyclin D1 **(J)**. Approximately 30% of the tumor cells show positive nuclear staining for C-Myc **(K)**. The Ki-67 proliferation index is approximately 90% **(L)** (IHC stain, all panels ×200; Scale bar: 100 µm).

Gross examination of the uterine cavity drainage fluid revealed 1.5 mL of red, turbid fluid. Liquid-based thin-layer cytology preparation revealed numerous lymphoid cells against a background of degeneration. These cells were characterized by large, hyperchromatic nuclei and scant basophilic cytoplasm. The nuclear contours were round, oval, or irregular (**Figure 3A**). At high-power magnification, the cells exhibited a high nuclear-to-cytoplasmic ratio, coarse chromatin, and occasional mitotic figures accompanied by prominent apoptosis (**Figure 3B**). These cytomorphologic features are consistent with lymphoma cells.

#### 3.5.2 Endometrial curettage histopathology

Gross examination revealed aggregated grayish-brown tissue fragments measuring 3.0 cm × 2.7 cm × 1.0 cm with medium consistency. Histologic examination revealed a diffuse lymphoid infiltrate set within a necrotic background, which exhibited a conspicuous starry-sky pattern (**Figure 3C**). The tumor cells were large and characterized by round, oval, or irregular nuclei. These cells possessed a high nuclear-to-cytoplasmic ratio, coarse chromatin, and prominent nucleoli (**Figure 3D**).

#### 3.5.3 Immunohistochemistry and *in situ* hybridization findings

The tumor cells were immunoreactive for CD20 (**Figure 3E**), PAX5 (**Figure 3F**), CD79α, CD5 (**Figure 3G**), BCL-6 (**Figure 3H**), and MUM1 (**Figure 3I**). They were negative for Cyclin D1 (**Figure 3J**), CD3, BCL2, CD30, and CD10. C-Myc positivity was observed in approximately 30% of tumor cells (**Figure 3K**), and the Ki-67 proliferation index was approximately 90% (**Figure 3L**). In situ hybridization for EBER was negative. The IHC findings were consistent with non-double-expressor DLBCL in this case. We further performed FISH analysis for *C-myc*, which showed no rearrangement, thus excluding double-hit lymphoma. The combined histological and IHC profile supports the diagnosis of CD5-positive diffuse large B-cell lymphoma, non-germinal center subtype.

### 3.6 Follow-up

The patient succumbed to the disease two weeks after diagnosis without having received lymphoma-specific therapy.

## 4 Discussion

Primary involvement of the female reproductive system by NHL is rare (0.2%-1.1% of extranodal cases) (5), with B-cell lymphoma, particularly DLBCL being the predominant type (6). The ovaries (38%) and cervix (20%) are the most common sites (7). Despite this predominance of DLBCL, its primary occurrence in the uterus is exceptionally rare, accounting for only about 14% of female reproductive system NHL (3, 4, 8).

Diagnosing primary NHL of the female reproductive system is challenging due to non-specific symptoms and overlapping features with common malignancies like endometrial carcinoma, often resulting in delayed diagnosis (3, 9). In the present case, the patient’s initial symptoms of abdominal distension and poor appetite, without vaginal bleeding, along with findings of persistent fever, cervical atrophy/adhesions, and suggestive imaging, led to an initial suspicion of endometrial carcinoma. The diagnosis remained elusive until cytological analysis of uterine cavity drainage fluid revealed lymphoma cells—a finding that was definitively confirmed as CD5-positive DLBCL on subsequent histopathological assessment of endometrial curettage specimens. The following findings confirmed its primary nature within the female reproductive system according to standard criteria (10): the patient presented with a uterine mass (evidenced by imaging and gynecological examination revealing an enlarged, firm uterus with massive fluid collection and cervical abnormalities) accompanied by regional lymph node involvement (mesenteric, retroperitoneal, bilateral iliac). Additionally, there was no peripheral blood or historical evidence of leukemia or prior lymphoma.

### 4.1 Clinical features of CD5-positive DLBCL

CD5-positive DLBCL represents a rare (5-10% of cases) and clinically aggressive immunophenotype, characterized by a five-year overall survival rate of approximately 40% (11, 12). A hallmark of this disease is its pronounced propensity for central nervous system (CNS) recurrence, which constitutes a major adverse prognostic factor; reported rates in primary disease are as high as 12.7-13.0% (2). The clinical profile typically involves elderly female patients and is distinguished by features such as elevated serum LDH and B symptoms (1). The current case concerned a 74-year-old female whose presentation—comprising recurrent high fever, weight loss, and marked elevations in LDH—aligned with this characteristic profile. Subsequent to diagnosis, however, imaging confirmation of CNS status was not obtained due to the patient’s decision to forgo further workup. Her clinical course was subsequently notable for rapid systemic deterioration accompanied by somnolence, a progression that raised considerable concern for potential CNS involvement.

### 4.2 Imaging features of uterine lymphoma

Uterine lymphoma, particularly DLBCL, demonstrates characteristic though often non-specific imaging findings across modalities. Sonographically, it typically presents as diffuse uterine enlargement with homogeneous, markedly hypoechoic masses that may mimic cystic changes; the endometrial line is often preserved. On contrast-enhanced ultrasound, these lesions may display a distinctive “starry-sky” enhancement pattern, significantly more intense than the adjacent myometrium (13, 14). On CT, the disease usually manifests as diffuse uterine enlargement with homogeneous soft-tissue attenuation masses that typically spare the endometrial and cervical epithelium. Associated lymphadenopathy may be present, while necrosis and cystic degeneration—common in other uterine malignancies—are notably absent (15).MRI offers the most discriminatory features. Lymphomatous tissue typically appears homogeneously iso- to hypointense on T1-weighted imaging and mildly hyperintense on T2-weighted imaging relative to the myometrium. The lesion’s high cellularity is reflected in marked hyperintensity on DWI due to restricted diffusion. Following contrast administration, moderate homogeneous enhancement is observed, consistent with the tumor’s hypervascularity and stromal proliferation (4, 16).

Despite these characteristic patterns, the non-specific appearance of uterine DLBCL on ultrasound and CT frequently leads to misdiagnosis. MRI, with its superior soft-tissue resolution, provides more definitive clues—as illustrated in the present case by the combination of T1 hypointensity, marked DWI hyperintensity, and patchy T2 hyperintensity. Ultimately, however, a definitive diagnosis of uterine lymphoma requires confirmation through histopathological examination.

### 4.3 Correlation between clinical and pathological features of CD5-positive DLBCL

DLBCL, not otherwise specified (NOS), represents the most common subtype among the various basic categories of DLBCL. However, only 5% to 10% of these cases may express CD5. CD5-positive DLBCL is currently recognized as an immunophenotypic variant rather than a distinct pathological entity (2, 17, 18). The diagnosis requires CD5 expression in more than 20% of neoplastic cells co-expressing B-cell markers such as CD20 or PAX5 (1). It is essential to distinguish primary CD5-positive DLBCL from cases transformed from lower-grade B-cell lymphomas, including chronic lymphocytic leukemia/small lymphocytic lymphoma (CLL/SLL) and mantle cell lymphoma (MCL) (2). And CD5-positive DLBCL, NOS, can be differentiated by the absence of cyclin D1 and SOX11 expression (18). In the present case, the histomorphological and IHC findings from the uterine curettage specimen supported the diagnosis of CD5-positive DLBCL, non-GCB subtype.

Among CD5-positive DLBCL cases, 76% display a centroblastic morphology, frequently featuring binucleated or “snowman-like” cells (approximately 85%) (2, 15, 19). Macroscopically, the uterine lesions often show diffuse growth while preserving the integrity of the endometrial or cervical epithelium. Consequently, cervical cytology is frequently negative; positive results typically suggest ulcer formation (16). In the present case, the uterine cavity drainage fluid contained numerous lymphoid cells with large, hyperchromatic nuclei and scant cytoplasm, demonstrating round, oval, or irregular nuclear contours, mitotic figures, and prominent apoptosis. The endometrial curettage specimens similarly revealed a diffuse lymphoid infiltrate with necrosis, consistent with the cytomorphological features described in the literature.

### 4.4 Diagnosis of CD5-positive DLBCL

The essential IHC markers for diagnosing CD5-positive DLBCL include CD5, CD20, PAX5, Ki-67, BCL2, MUM1, and CD10. In the present case, an extended panel (CD79α, CD19, Bcl6, CD3, CyclinD1, C-Myc, CD30, EBER) was also evaluated. The diagnostic and prognostic significance of these key markers is summarize in **Table 1** (1, 2, 20, 21).

**Table 1:** Diagnostic and prognostic significance of key immunohistochemical markators in CD5-positive DLBCL.

Marker	Definition / characteristics	Clinical significance
CD5	A 67kDa transmembrane glycoprotein; a defining marker for this DLBCL subtype.	Positivity (membranous expression in >20% of neoplastic B-cells) correlates with a poorer prognosis (1).
CD20	A B-cell lineage-specific surface marker involved in B-cell activation and proliferation.	A widely used pan-B-cell marker. Its loss may indicate plasmablastic differentiation (15).
PAX5	A transcription factor critical for B-cell development and commitment, expressed from pro-B to mature B-cell stages.	A highly reliable nuclear marker for B-cell lineage. Often co-expressed with CD20 and CD79α in B-cell lymphomas (15).
Ki-67	A nuclear protein that serves as a key marker for cellular proliferation.	A high proliferation index (>60%) is typical for DLBCL. CD5-positive cases frequently show a very high index (>90%), indicating heightened aggressiveness (2, 15).
BCL2	A 25kDa mitochondrial membrane protein that functions as a key inhibitor of apoptosis.	High frequency of protein expression (>70%) in CD5-positive DLBCL is independently associated with a poor prognosis (2).
MUM1/IRF4	A lymphocyte-specific transcription factor expressed during late germinal center differentiation and plasma cell development.	Common expression supports the classification of CD5-positive DLBCL as a non-germinal center B-cell (non-GCB) subtype (20).
CD10	A cell surface metalloprotease expressed on germinal center B-cells.	CD5-positive DLBCL is typically CD10-negative, classifying it as a non-GCB subtype, which is generally associated with a poorer prognosis (20).
Cyclin D1	A key cell cycle regulator, serving as a diagnostic marker for mantle cell lymphoma.	Primarily used to exclude mantle cell lymphoma. Over 90% of primary CD5-positive DLBCL cases express Cyclin D2 instead (21).
C-Myc	A potent oncoprotein that regulates cell proliferation and metabolism.	Expression (seen in 30-50% of DLBCL NOS) is often evaluated in the context of "double expressor" status (with BCL2) and can be associated with B symptoms and aggressive disease.
BCL6	A transcription factor that is a key marker for germinal center B-cells and their derived lymphomas.	Its expression pattern aids in cell-of-origin classification. The prognostic impact of its rearrangements in CD5-positive DLBCL is less clear compared to CD5-negative cases.

A definitive diagnosis requires strict adherence to the immunophenotypic criterion of >20% CD5 membrane positivity in neoplastic B-cells. Imaging features, such as diffuse uterine enlargement with preserved endometrial integrity and characteristic MRI findings (e.g., homogeneous signal intensity and enhancement patterns), when coupled with elevated serum LDH, can raise early suspicion for uterine CD5-positive DLBCL and prompt timely histopathological evaluation. In cases where an initial tissue biopsy is challenging, as encountered here, cytological examination of uterine cavity drainage fluid offers a valuable diagnostic alternative. If the sample is sufficient, cell block preparation followed by staining for key IHC markers can reliably establish the diagnosis.

### 4.5 Treatment and prognosis of CD5-positive DLBCL

The first-line treatment for CD5-positive DLBCL is the R-CHOP regimen (rituximab, cyclophosphamide, doxorubicin, vincristine, and prednisone) (22). Surgical intervention is primarily indicated for obtaining diagnostic tissue rather than achieving radical resection, and extensive procedures should be avoided (23). Given the elevated risk of CNS recurrence compared to ovarian DLBCL, intrathecal prophylaxis is recommended when indicated to mitigate this risk (3). Radiotherapy may be considered for managing residual disease or involved lymph node regions (24).

CD5-positive DLBCL tends to be associated with a poorer prognosis, a finding that is more pronounced in Asian patients (18), and is more frequently of the activated B-cell (ABC) subtype (71%–82%). In contrast, the GCB subtype of primary CD5-positive DLBCL does not show a significant prognostic difference compared with CD5-negative DLBCL (25, 26).This case concurrently exhibited a non-double-expressor DLBCL phenotype. Compared with double-expressor lymphoma, non-double-expressor DLBCL exhibits distinct genetic alterations and signaling pathway profiles (27), and is generally associated with a more favorable prognosis (28, 29). However, in CD5-positive DLBCL cases, the expression rates for BCL-2, BCL-6, and C-Myc were 71.86%, 60.10%, and 36.68%, respectively. Notably, the prevalence of the double-expressor phenotype was 48.17% (1, 15, 19, 30). In the aforementioned case study, the prognosis of CD5-positive DLBCL, compared to CD5-negative cases, does not show a significant correlation with C-Myc, BCL2, and/or BCL6 rearrangements (1, 30). The prognostic significance of uterine DLBCL continues to be discussed. Although some studies suggest a more favorable overall survival for affected patients managed with R-CHOP relative to their non-uterine counterparts (31), other investigations have reported an inferior prognosis (32). The latter may be explained by delays in establishing the diagnosis. It is therefore imperative to heighten awareness of this disease among clinicians and within diagnostic departments to ensure timely diagnosis and achieve optimal therapeutic outcomes.

### 4.6 Strengths and limitations

This case report highlights the diagnostic utility of uterine cavity drainage fluid cytology in an elderly patient where initial tissue biopsy was precluded by cervical atrophy and adhesions. The integrated analysis of clinical, laboratory, imaging, and cytological findings provided crucial diagnostic clues, subsequently confirmed by histopathology. This approach offers a valuable reference for similar challenging cases where tissue acquisition is not immediately feasible.

However, the patient’s decision to forego further intervention following diagnosis precluded comprehensive staging, lymphoma-specific therapy, and consequently, assessment of treatment response and long-term prognosis. Additionally, the limited specimen, compounded by extensive necrosis, prevented ancillary studies such as cell block preparation for extended IHC. These limitations underscore the inherent challenges in diagnosing and managing rare entities like uterine CD5-positive DLBCL, particularly in resource-limited or clinically complex settings.

## 5 Conclusion

Uterine CD5-positive DLBCL is a rare and highly aggressive neoplasm whose diagnosis relies on histopathology and IHC. Enhancing awareness of this entity among clinicians and pathologist is crucial for facilitating early diagnosis and optimizing therapeutic outcomes.

## Data Availability

The original contributions presented in the study are included in the article/supplementary material. Further inquiries can be directed to the corresponding author.
